# Generation and Characterization of Mice Carrying a Conditional Allele of the *Wwox* Tumor Suppressor Gene

**DOI:** 10.1371/journal.pone.0007775

**Published:** 2009-11-10

**Authors:** John H. Ludes-Meyers, Hyunsuk Kil, Jan Parker-Thornburg, Donna F. Kusewitt, Mark T. Bedford, C. Marcelo Aldaz

**Affiliations:** 1 Department of Carcinogenesis, University of Texas M.D. Anderson Cancer Center, Science Park-Research Division, Smithville, Texas, United States of America; 2 Department of Biochemistry and Molecular Biology, University of Texas M.D. Anderson Cancer Center, Houston, Texas, United States of America; School of Medicine, University of Barcelona, Spain

## Abstract

*WWOX*, the gene that spans the second most common human chromosomal fragile site, FRA16D, is inactivated in multiple human cancers and behaves as a suppressor of tumor growth. Since we are interested in understanding *WWOX* function in both normal and cancer tissues we generated mice harboring a conditional *Wwox* allele by flanking Exon 1 of the *Wwox* gene with LoxP sites. *Wwox* knockout (KO) mice were developed by breeding with transgenic mice carrying the *Cre*-recombinase gene under the control of the adenovirus *EIIA* promoter. We found that *Wwox* KO mice suffered from severe metabolic defect(s) resulting in growth retardation and all mice died by 3 wk of age. All *Wwox* KO mice displayed significant hypocapnia suggesting a state of metabolic acidosis. This finding and the known high expression of *Wwox* in kidney tubules suggest a role for *Wwox* in acid/base balance. Importantly, *Wwox* KO mice displayed histopathological and hematological signs of impaired hematopoeisis, leukopenia, and splenic atrophy. Impaired hematopoeisis can also be a contributing factor to metabolic acidosis and death. Hypoglycemia and hypocalcemia was also observed affecting the KO mice. In addition, bone metabolic defects were evident in *Wwox* KO mice. Bones were smaller and thinner having reduced bone volume as a consequence of a defect in mineralization. No evidence of spontaneous neoplasia was observed in *Wwox* KO mice. We have generated a new mouse model to inactivate the *Wwox* tumor suppressor gene conditionally. This will greatly facilitate the functional analysis of *Wwox* in adult mice and will allow investigating neoplastic transformation in specific target tissues.

## Introduction

WW domain-containing oxidoreductase (*WWOX*) was cloned and identified as a potential tumor suppressor gene mapping to the chromosome region 16q23 [1]. The *WWOX* gene spans >1.1 Mb and overlaps the common chromosomal fragile site, FRA16D, the second most common site for chromosomal breakage, instability and rearrangement of the whole genome [1]–[3]. Allelic losses and rearrangements affecting the *WWOX* locus have been described in various human cancers [1]–[5]. Additionally, various studies have reported significant loss of *WWOX* expression in multiple human neoplasias including breast, prostate, ovarian, lung and liver cancer [6], [7].

WWOX is a 46-kD protein, highly conserved through evolution, that contains two N-terminal WW-domains and a short chain dehydrogenase/reductase domain (SDR domain) [1]. Our understanding of the NAD(P)(H)-dependent enzymatic functions of WWOX is very limited. WWOX's structure is the archetypal representative of one of four separate clusters of classical SDRs conserved through evolution [8], [9]. Based on the high expression of WWOX in hormonally regulated tissues (testis, prostate and ovary) and its amino acid sequence homology to specific oxidoreductases, we postulated that WWOX maybe an enzyme involved in sex-steroid metabolism [1]. The WW-domains are required for protein-protein interactions through binding the conserved proline-rich sequence PPXY. Several proteins have been identified as WWOX protein partners such as SIMPLE, SCOTIN [10] and EZRIN [11] as well as several transcription factors such as the tumor suppressor p73[12], ERBB4-CTF [13], AP2γ [14], MET-CTF [15] and the osteoblast differentiation master regulator, RUNX2 [16], to name a few. One role for WWOX has emerged as a regulator of transcription by limiting transcription factor access to the nucleus through cytoplasmic sequestration [6].

The role of *WWOX* as a tumor suppressor is supported by *in vitro* studies with human cancer cells and *in vivo* studies using mouse models. Several studies reported that ectopic WWOX expression suppressed the *in vivo* tumorigenicity of various human tumor types including, breast [5], lung [17], prostate [18] and ovarian [19] cancer cells when xenografted in nude mice. Mouse models with targeted disruption of *Wwox* had increased spontaneous tumor incidence. Using a conventional mouse knockout approach Aqeilan et al. [20] reported development of osteosarcomas in some *Wwox^−/−^* mice by the age of 2.5 weeks and as early as postnatal day 3. All *Wwox* null mice died by 3–4 wk of age precluding any further studies of adult animals. However, development of spontaneous lung papillary carcinoma was observed in adult *Wwox^+/−^* mice. Aqelian et al [20] also reported that *Wwox^+/−^* mice treated with chemical carcinogens displayed an increased incidence of lymphomas and lung tumors when compared with wild type littermates. Using a gene trap approach we developed a *Wwox* hypomorphic mouse model [21]. Female *Wwox* hypomorphs had an increased incidence of spontaneously arising B-cell lymphomas. In addition, several female developed multiple neoplasias. Together, the in vitro and in vivo studies have provided significant evidence for *Wwox* as a tumor suppressor.

We are interested in understanding in more detail the normal and tumor suppressive role *Wwox* plays in the multiple tissues it is expressed. As mentioned, *Wwox* KO mice generated using conventional techniques die by four weeks of age thus impeding any studies in adult tissues. Therefore, we report here the generation of mice with a conditional allele for *Wwox* ablation for better understanding the roles of *Wwox* in different tissues and in pathological conditions.

## Results

### Generation of Mice with a Floxed-*Wwox* Allele

**Figure 1:**
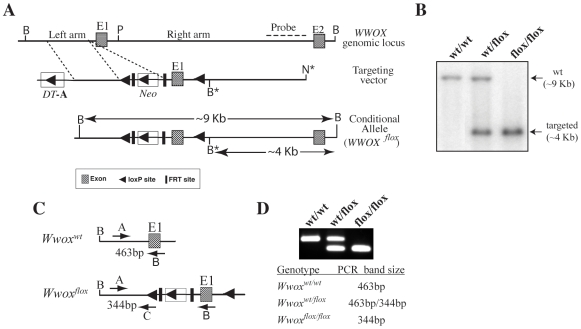
Targeting the Mouse *Wwox* allele. **(A)** Insertion of the targeting vector into the *Wwox* allele introduced a new Bgl II (B) restriction site into *Wwox* Intron 1. The new Bgl II site leads to a smaller Bgl II restriction fragment when analyzed by Southern blot with the external hybridization probe. Additionally, a Not I restriction site was engineered at the 3′ end of the right arm used to linearize the targeting construct prior to electroporation. **(B)** Southern blot genotyping of wild-type (wt/wt) mice, heterozygous mice (wt/flox) and homozygous mice (flox/flox). **(C)** Multiplex-PCR based genotyping using allele specific primers. Primers A and B anneal to *Wwox* specific sequences in the *Wwox* upstream regulatory region (primer A) and in *Wwox* Exon 1 (primer B). Primer C anneals to sequences in the LoxP *Cre*-recombination in the targeted *Wwox* allele. Multiplex PCR using primers A, B and C yield DNA products having sizes specific for the wild-type and targeted alleles.

We devised a strategy for conditional ablation of *Wwox* gene expression using the *Cre*-Lox site-specific recombination system. Our approach, illustrated in Figure 1, utilized positive-negative selection gene targeting [22] to modify *Wwox* genomic sequences by flanking *Wwox* Exon 1 with two LoxP-recombination sites to generate a “floxed”-*Wwox* allele. We successfully targeted mouse ES cells and generated chimeric founders having germline transmission and subsequently bred a *Wwox^flox^* mouse.

Since it was possible that the inserted *pgk-neo* plasmid could impair *Wwox* gene transcription we determined whether the floxed-*Wwox* allele had any effect on viability and fertility. Breeding of heterozygous (*Wwox^+/flox^*) mice from two different founders resulted in pups having genotypes with the expected Mendelian ratios demonstrating that the floxed allele had no effect on embryonic development or viability. Mating of *Wwox^flox/flox^* males with *Wwox^flox/flox^* females resulted in normal litter size, genotype frequencies and pups had normal postnatal development. These results showed that the “floxed”-*Wwox* allele containing the insertion of the ∼2 Kb neomycin resistance gene in the 5′ transcriptional control region of the *Wwox* gene had no detrimental affect on *Wwox* functions required for normal mouse growth, survival or reproduction. Therefore, this mouse model will be a useful tool for analyzing *Wwox* function(s) in specific mouse tissues.

### *Cre*-Recombinase Deletion of the *Wwox* Gene Completely Ablates *Wwox* Expression

**Figure 2:**
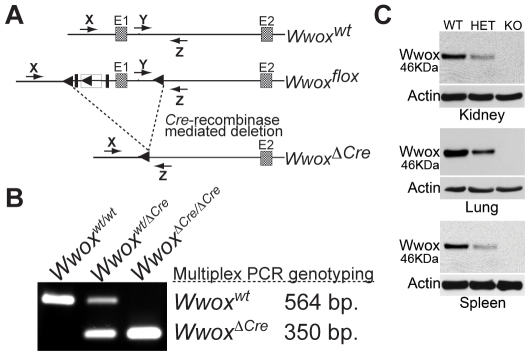
Strategy for *Cre*-mediated ablation of *Wwox* expression. **(A)** To promote deletion of *Wwox^flox^* alleles we used transgenic mice carrying the *Cre*-recombinase gene under control of the adenoviral *EIIA*-promoter. *EIIA*-regulated *Cre*-recombinase is expressed in pre-implantation embryos leading to site-specific deletion of LoxP flanked (floxed) sequences in all tissues including germ cells. **(B)** PCR-based strategy to demonstrate *Cre*-recombinase deletion of the *Wwox* target sequences. **(C)** Wwox protein expression is abolished in *Wwox* KO mice. Total protein extracts from the indicated tissues were analyzed by immunoblotting using Wwox specific antibodies. Anti-actin was used as a loading control. WT-wild-type, HET-heterozygous, KO-knockout.

We showed that our strategy resulted in the specific modification of the *Wwox* allele resulting in the insertion of the two LoxP-recombination sequences flanking Exon 1 however it is possible that small changes to the inserted sequence could have been introduced during the procedure. Therefore, to determine whether *Cre*-recombinase could utilize the inserted LoxP sites to delete *Wwox* Exon 1, we generated mice combining the *Wwox^flox^* allele with the Cre-recombinase gene under the control of the adenovirus *EIIA* promoter. The adenovirus *EIIA* promoter directs transcription of *Cre*-recombinase at the zygote stage of embryonic development resulting in deletion of floxed sequences in all cells of the developing embryo including the germ cells [23]. To determine whether *Cre*-recombination was successful we analyzed genomic DNA using PCR with oligonucleotide primers specific for wild-type or *Cre*-deleted *Wwox* sequences (Figure 2). Using this strategy we were able to test the functionality of the LoxP sites, determine whether our strategy of deleting Exon 1 would eliminate *Wwox* expression and give rise to a phenotype.

We then mated *Wwox^flox/flox^* mice with homozygous *EIIA-Cre* transgenic mice that resulted in the first familial generation (F_1_) of pups having one *Wwox* allele with wild-type sequences and one *Wwox* allele with Exon 1 deleted (*Wwox^+/ΔCre^*). Since the *Wwox^ΔCre^* allele was present in the germ cells a F_1_×F_1_ cross should result in pups having all possible genotypes (*Wwox^+/+^*; *Wwox^+/ΔCre^*; *Wwox^ΔCre/ΔCre^*) with Mendelian ratios of 1∶2∶1 (Table 1). Successful matings from all mating pairs (n = 10) were obtained demonstrating that Wwox^+/ΔCre^ mice were fertile having litter sizes ranging between 3 to 11 pups (average  = 7.75). Three-day old pups were genotyped by PCR (Figure 2) and correct Mendelian ratios were obtained after genotyping 154 animals (p = 0.257 using the χ^2^-test). To demonstrate that our targeting strategy lead to complete ablation of *Wwox* expression we performed immunoblot analysis of total protein extracts from a variety of tissues (Figure 2). As expected Wwox protein was undetectable in *Wwox^ΔCre/ΔCre^* mice and was reduced by approximately one-half in heterozygotes confirming that *Cre*-mediated deletion of *Wwox* Exon 1 lead to the complete absence of Wwox protein.

**Table 1:** Genotypes of pups from *Wwox^+/ΔCre^* × *Wwox^+/ΔCre^* crosses.

Genotype	WT	HET	KO	Total
Actual # of pups	39	85	30	154
Expected # of pups	38.5	77	38.5	154

**Figure 3:**
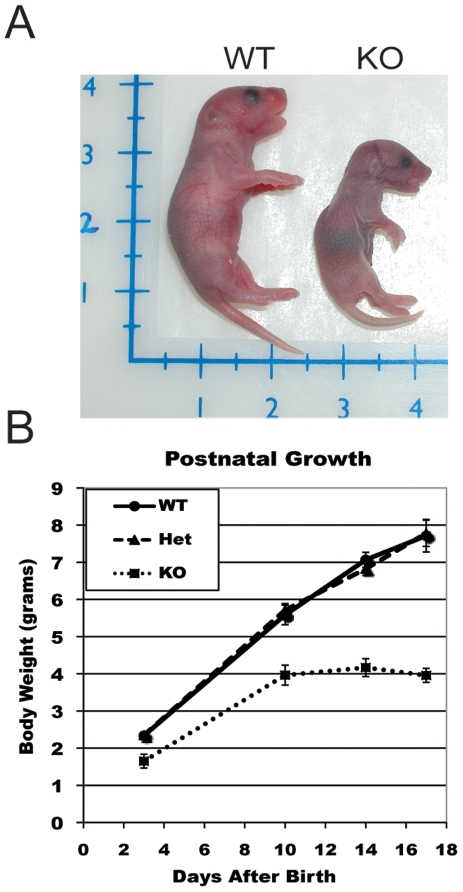
*Wwox* KO mice have reduced postnatal growth. **(A)** Photograph of WT and KO newborn littermates. **(B)** F2 pups were weighed on postnatal days 3, 10, 14 and 17. 100% of *Wwox* KO mice died before weaning (21 days). WT, n = 18; HET, n = 43; KO, n = 8. Error bars represent ±SEM.

Ablation of *Wwox* expression lead to significant growth retardation that was noticeable at birth (Figure 3A). To further address pup growth we weighed pups periodically and found that *Wwox* KO mice had a severe growth defect averaging only 4.2 g at postnatal day 14 compared to heterozygous and wild-type mice (6.82 and 7.07 g, respectively). Although some *Wwox* KO mice can survive up to 3 weeks of age, the mortality rate was very high. As early as 72 h after birth 43% (15 of 35) of *Wwox* KOs had died and 77% had died by 17 days after birth and no mice survived past weaning. These findings demonstrated that ablation of *Wwox* expression by deleting Exon 1 using our *Cre*-loxP strategy lead to growth retardation and postnatal death in agreement with the observations of Aqeilan et al [16], [20] using conventional knockout technology.

### Ablation of *Wwox* Expression by *EIIA-Cre* Mediated Deletion Resulted in Impaired Hematopoiesis and Splenic Athrophy

**Figure 4:**
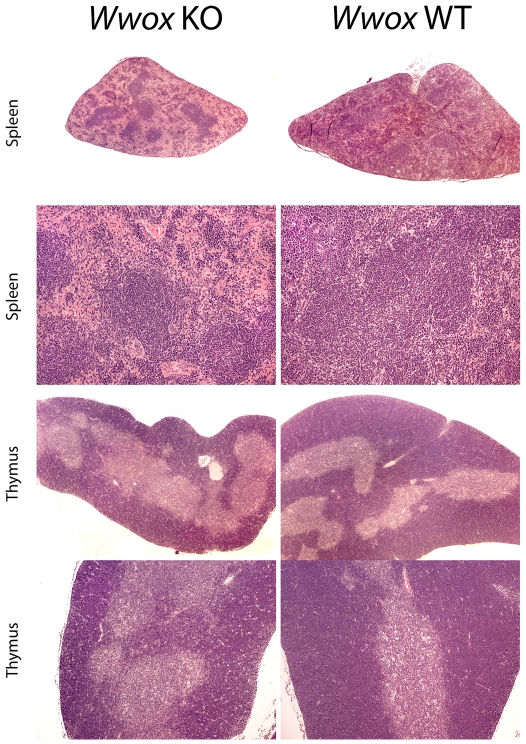
*Wwox* KO mice have abnormal spleens and thymuses. Histopathology of spleens (top four panels) and thymuses (bottom four panels) from 18 day old KO and WT mice. Histological sections were stained with H&E. Note the significant atrophy of the spleen and the reduced cortical thickness in the thymus from the KO mice. Low power images of spleens (first row) and thymuses (third row) have a total magnification of 21X. High power images of spleens in (second row) have a total magnification of 84X and thymuses (bottom row) have a total magnification of 44X.

To try to understand the cause of death of *Wwox* KO mice we performed necropsies and analyzed blood chemistries of mice at postnatal day 18. Necropsy results found significantly reduced relative weights of multiple organs and increased brain weight in KO mice consistent with wasting (Table 2). Interestingly, spleens of KO mice were dramatically reduced in size and weight when compared with those of wild-type and heterozygous. The average *Wwox* KO spleen weighed 9.3 mg on average, representing 0.21% of whole body weight, while spleens from wild-type and heterozygotes mice weighed on average 4 times as much representing up to 0.53–0.46% of whole body weight, i.e more than 2 fold that relative weight of KO mice (p = 0.0015) (Table 2). The histology of the *Wwox* KO mice spleens was very abnormal displaying signs of spleen atrophy. As can be observed in Figure 4, the red pulp displayed dramatically reduced cellularity, although hematopoietic cells of all series appear to be present. Lymphoid aggregates of the white pulp of the KO spleens were also reduced in size compared to the WT spleens.

**Table 2:** Organ Weights.

Organ	WT (n = 3)	HET (n = 3)	KO (n = 3)	p-value***
Spleen (mg)	39.4*	33.3	9.30	0.0015
	0.53(±0.03)% **	0.46(±0.06)%	0.21(±0.03)%	
Liver (g)	0.320	0.279	0.144	0.0367
	4.0(±0.15)%	3.9(±0.07)%	3.4(±0.29)%	
Kidney (g)	0.120	0.110	0.059	NS
	1.5(±0.03)%	1.5(±0.06)%	1.4(±0.06)%	
Adrenal (mg)	2.27	1.67	2.97	NS
	0.03(0.003)%	0.02(±0.006)%	0.06(±0.029)%	
Thymus (mg)	60.0	55.0	25.1	0.0495
	0.77(±0.05)%	0.77(±0.03)%	0.58(±0.10)%	
Heart	0.063	0.056	0.034	NS
	0.79(±0.02)%	0.78(±0.06)%	0.80(±0.03)%	
Brain	0.390	0.371	0.356	0.0003
	5.0(±0.31)%	5.2(±0.28)%	8.5(±0.65)%	

Organ weights are given as average *absolute weight or **absolute weight/organ weight as a % of body weight (±SEM).

***p-value using student's t-test comparing WT+HET vs. KO; NS-p>0.05.

We also compared the histology of thymus that also displayed reduced relative weight (p = 0.05). We observed that in spite of preserving relatively normal organ architecture, the cortex of the thymus of KO mice is significantly thinner than that of wild type counterparts (Figure 4).

Given these histopathological findings we proceeded to perform some hematological analyses. White blood counts (WBC) were estimated manually on blood smears obtained from mice of the different genotypes. WBC for wild-type mice was estimated at an average of 9.45×10^3^ cells *per* ul (±2.25, n = 2) while *Wwox* KO mice showed signs of leukopenia with lower WBC at an average of 4.2×10^3^ cells *per* ul (±1.20, n = 2). No major differences were observed in the relative representation of the various white blood cell subtypes (not shown). However, one of the two KO mice analyzed displayed 3% of nucleated red blood cells (NRBC)/100 WBCs, a clear sign of severe anemia, while none of the WT mice showed any NRBCs. In summary, the histopathological and hematological findings suggest significantly impaired hematopoiesis in *Wwox* KO mice affecting white and red blood cell lineages.

### *Wwox* KO Mice Displayed Signs of Metabolic Acidosis and Kidney Failure

Most of the parameters measured by blood chemistry were not significantly different in the *Wwox* KOs compared with wild-type and heterozygotes (Table 3). However, glucose levels of *Wwox* KO mice were significantly lower than wild-type and heterozygotes (63% of HET and 57% of WT levels, Table 3). Circulating calcium levels were also significantly lower in KO mice (p = 0.0004). More importantly, *Wwox* KO animals displayed blood chemistry values compatible with kidney failure since they showed higher blood urea nitrogen (BUN) (two-fold higher, p = 0.01). In addition, KO mice displayed dramatically reduced bicarbonate levels (CO_2_) in blood (p = 0.006) indicative of serious disturbance in the acid/base balance compatible with metabolic acidosis.

**Table 3:** Blood Chemistry Analysis.

Test	WT (n = 3)	HET (n = 3)	KO (n = 4)	p-value**
BUN (mg/dL)	17.67±2.60*	18.33±1.20	37.25±5.73	0.01086
Glucose (mg/dL)	250.6±5.04	227.0±11.7	143.50±8.81	0.000131
Total Protein (g/dL)	4.200±0.058	4.10±0.000	4.10±0.252	NS
Albumin (g/dL)	2.70±0.000	2.63±0.067	2.60±0.200	NS
Globulin (g/dL)	1.50±0.580	1.47±0.067	1.50±0.058	NS
Calcium (mg/dL)	11.13±0.120	10.87±0.120	10.18±0.295	0.000385
Phosphorus (mg/dL)	12.60±0.306	12.37±0.318	12.73±0.544	NS
Sodium (Eq/L)	143.00±0.577	143.67±0.667	142.00±6.00	NS
Potassium (mEq/L)	8.97±0.219	8.90±0.252	9.90±1.9	NS
Chloride (mEq/L)	108.67±0.667	108.67±1.20	112.50±1.50	0.02769
Total CO_2_ (mEq/L)	21.67±0.333	21.33±0.333	14.50±3.5	0.006227
Osmolality (mOsmo/kg)	306.23±0.517	306.50±1.55	304.75±14.2	NS

*Values are presented as the average±SEM.

**p-value using student's t-test comparing WT+HET vs. KO; NS-p>0.05.

### *Wwox* is Required for Normal Bone Mineralization

**Figure 5:**
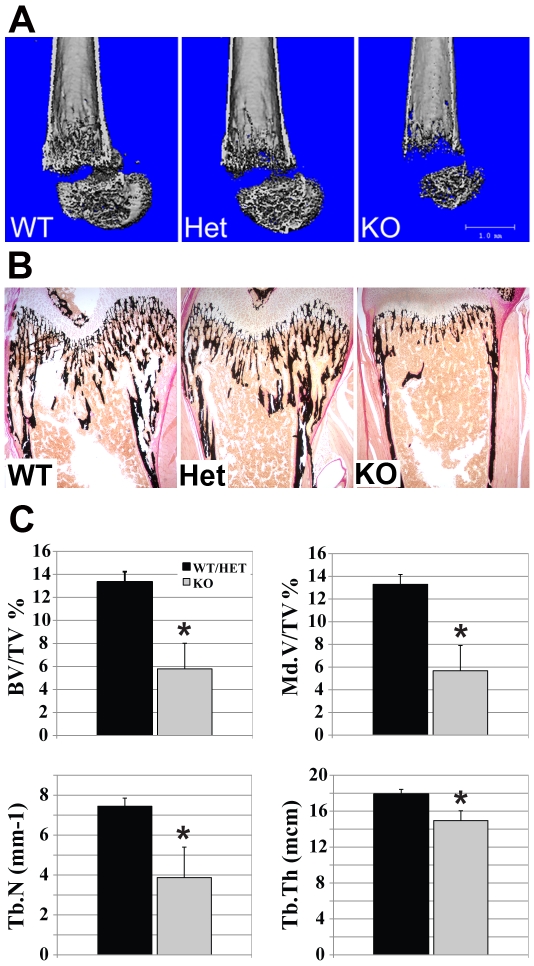
Histomorphometric analysis. **(A)** MicroCT images of femurs from postnatal day 18 mice. Note the dramatic difference in bone structure of the femur from KO mice vs WT and HET mice bones **(B)** Von Kossa stained sections of femurs from mice of the indicated genotypes. Black stained areas represent mineralized bone tissue. Note the significantly decreased mineralization in the KO mice bone sample **(C)** Quantitative histomorphometric analyses of postnatal day 18 day mice. We did not observe differences between wild-type and heterozygous mice, therefore, we compared KO data (n = 3) with WT and HET data combined (WT+HET, n = 5). Wild-type-WT, heterozygote-HET, knockout-KO. BV/TV%-bone volume/tissue volume, Md.V/TV%-mineralized volume/tissue volume, Tb.N-Trabecular number (1/mm), Tb.Th-Trabecular thickness (micrometers). *p<0.05.

Since the KO mice were markedly smaller in size compared to litter mates and it was previously reported that *Wwox* KO mice had defects in bone formation [16] we decided to perform bone analyses of our *Wwox* KO mouse. We used micro-computed tomography (micro-cT) and bone histomorphometry to measure several parameters of endochondral bone formation. Femurs of 18-day old mice were used for these analyses. Micro-cT analyses were used to obtain high-resolution scans and constructed 3-D images to examine morphological features (Figure 5A). We observed a significant decrease in bone volume and cortical thickness in *Wwox* KO mice compared to WT and HETs. Also, it was evident that cortical porosity and the number of trabeculae were decreased in *Wwox* KO mice. We also subjected femurs of the same mice to detailed histomorphometric analyses. For these studies sections of femurs were quantified histologically for static parameters of bone formation such as bone volume, trabecular number and cell counts. Sections of femurs stained using Von Kossa that identifies mineralized bone clearly demonstrated a reduction of bone formation in KO mice (Fig. 5B). The most significant difference observed in *Wwox* KO femurs was in bone volume. Bone volume measurements quantify the volume of mineralized and non-mineralized portions (osteoid) of the tissue. The bone volumes of femurs from KO mice were reduced >66% in comparison to femurs from WT and HET mice (BV/TV %, 13.35% vs. 5.8%, respectively; p<0.01, Fig. 5C). To more clearly define which parameters of bone formation were impaired in KO mice, volumes of mineralized and osteoid tissue were determined. Interestingly, femurs of KO mice were significantly less mineralized compared to WT and HET mice (Md.V/TV %, 13.3% vs. 5.7%, p<0.01, Fig. 5C) and KO mice femurs tended to have more osteoid than WT and HET femurs (OV/BV %, 0.36% vs. 3.5%, p = 0.07). The number and thickness of trabeculae were also reduced in femurs of KO mice (Fig. 5C). This was also observed using micro-cT. This indicates that there was a decrease in bone formation in *Wwox* KO mice that is likely due to a lack of mineralized tissue.

Therefore, to understand the defect of bone formation in *Wwox* KO mice we performed histomorphometric measurements of osteoblast and osteoclast activity. Measurements of osteoclast activity did not show any differences in femurs from mice of any genotype. We quantitated osteoblast activity and found that there were no significant differences in the number of active and non-active osteoblasts in femurs from all genotypes. Additionally, osteoblasts of KO mice were healthy, plump, cuboidal cells of normal morphology. However, there was a significant difference in the number of active osteoblasts over bone surface (N. Ob/BS) in femurs of KO mice (p = 0.02). This indicates that osteoblasts of *Wwox* KO have a defect in mineralization resulting in reduced bone formation.

A total of nine 18d old *Wwox* KO mice were subjected to complete necropsies, X ray analyses and multiple organ histopathology. We did not observed any lesions compatible with neoplastic growth by any of the methodologies. Importantly, X ray analyses did not reveal any bone lesions compatible with osteosarcoma.

## Discussion

The *WWOX* tumor suppressor gene is inactivated in many types of human cancers by genomic rearrangements, aberrant mRNA splicing, homozygous deletions and epigenetic silencing [1]–[5], [24]. Because *Wwox* KO mice do not survive to adulthood previous studies of *Wwox* tumor suppression required chemical carcinogen treatment of *Wwox^+/−^* mice [20]. Therefore, we have generated mice carrying a conditional allele of the *Wwox* tumor suppressor gene.

In this report we describe the generation and initial characterization of a novel mouse model with a conditional *Wwox* allele. We utilized adenovirus *EIIA*-regulated *Cre*-recombinase transgenic mice to delete *Wwox* Exon 1 in all mouse tissues and ultimately generated *Wwox* KO mice. We found that *Wwox* KO mice suffered from severe metabolic defect(s) resulting in significant growth retardation, hypoglycemia, impaired hematopoeisis, and signs of metabolic acidosis that ultimately lead to premature death of all mice by 3 weeks of age. Bone formation of *Wwox* KO was also significantly affected. Bones from KO mice were analyzed and found to be smaller and thinner having reduced bone volume as a consequence of a defect in mineralization.

**Figure 6:**
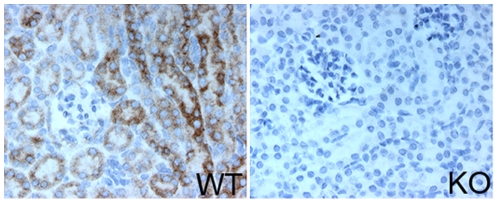
*Wwox* is strongly expressed in the kidney tubules. Kidneys from a WT (left panel) or a KO (right panel) 18 day old mouse were dissected, paraffin embedded and subjected to immunohistochemical staining using anti-WWOX antibody. The strongest staining structures represent the distal convoluted tubule section of nephrons. Photomicrographs were taken using a 40X objective.

The results of the blood chemistry analysis demonstrated that *Wwox* KO mice had severe metabolic defects. Hypoglycemia can result from multiple causes such as disorders in carbohydrate, fat or amino acid metabolism as well as endocrine system disorders. Since *Wwox* is expressed in a variety of endocrine, neuroendocrine and non-endocrine tissues it is not possible from these studies to identify the cause of hypoglycemia. The combination of increased BUN with reduced bicarbonate levels (hypocapnia) suggests impaired kidney function and a disturbance in the blood acid-base balance that would result in acidosis. Protracted acidosis disturbs many bodily functions and can lead to growth retardation, bone disease, possibly total kidney failure and ultimately death. Renal tubular acidosis (RTA) is a pathology that occurs when the kidneys fail to excrete acids into the urine, which causes the blood to remain too acidic due to defects of cells in the proximal and distal convoluted tubules. The convoluted tubules of healthy kidneys help maintain acid-base balance by excreting acids into the urine and returning bicarbonate to the blood. Interestingly, we have previously demonstrated that *Wwox* is highly expressed in distal convoluted tubules and to a lesser extent in the proximal tubules of the kidney in mice (Figure 6) and humans [25]. Given the pathology observed and the location of *Wwox* expression in the kidney we hypothesize that WWOX may play a role in the regulation of blood pH and maintenance of electrolyte balance. Thus, we speculate that the lack of *Wwox* expression in the kidney tubules in KO mice is likely responsible for the development of severe metabolic acidosis of renal tubule origin (RTA) that ultimately leads to death. In addition, as significant contributing factors to morbidity and death, we also observed severe histopathological abnormalities affecting hematopoietic organs such as spleen and thymus in addition to white blood cell counts compatible with a significant suppression on hematopoiesis affecting the *Wwox* KO mice. Specifically, the spleens of *Wwox* KO mice displayed signs of atrophy with dramatic reduction in red pulp cellularity. All studies indicate that *Wwox* KO mice suffered from anemia and leukopenia. Anemia can be also intimately linked to kidney function and to metabolic acidosis. Additional studies will be required to determine the exact causes of the impaired hematopoiesis observed as a result of *Wwox* ablation.

*Wwox* KO mice have been described to suffer alterations in bone metabolism [16]. We developed this conditional mouse model to facilitate the exploration of the cell autonomous function(s) of *Wwox* that contribute to the variety of abnormalities we and others have observed. Therefore, we wanted to determine whether our mouse model of *EIIA-Cre* mediated ablation of *Wwox* expression recapitulated the observed defects in normal mouse bone development. We used micro-cT and histomorphometry to measure several static bone parameters. We observed a decrease of bone formation in *Wwox* KO mice associated with a reduction of mineralization and concomitant increase in osteoid compared to WT and HET mice. Proper bone formation results from a fine balance between bone formation and bone resorption. This balance is mostly maintained by the activity of two bone cell types, osteoblasts and osteoclasts, that make bone and degrade bone respectively. We measured osteoblast activity and found no significant differences in the number of active and non-active osteoblasts in femurs between genotypes. Measurements of osteoclast activity did not show any differences in femurs from mice of any genotype either. This is in sharp contrast to the observations of Aqeilan et al [16] who observed consistently higher numbers of osteoclast activity in bone sections of KO mice however they did not perform quantitative analyses in those studies. We conclude that *Wwox* KO mice have reduced bone formation due to a bone mineralization defect. This is consistent with previous results that demonstrated a significant defect in differentiation of *Wwox* KO calvarial osteoblasts ex vivo compared to WT osteoblasts [16]. Notably, heterozygous mice did not show any abnormalities in postnatal growth and survival as well as blood or bone parameters highlighting that only one *Wwox* allele is sufficient for full function.

Importantly, Aqeilan et al. also reported that 4 of 13 (31%) mice *Wwox* KO mice developed focal lesions along the femoral diaphysis compatible with periostal osteosarcoma, lesions observed in one mice as early as 3d of age [20]. It is worth noting that in contrast with those previous observations, we did not detect any lesions compatible with spontaneous osteosarcomas in any of the *Wwox* KO mice analyzed macroscopically, histopathologically and radiographically. Furthermore, none of the long bones that were subjected to MicroCT analyses revealed any malignant lesions either. The reason(s) for the discrepancies between studies remain to be determined.

Very recently a spontaneous mutation in the *Wwox* gene was identified in rats that showed severe growth retardation, experienced epileptic seizures and died without reaching maturity [26]. The mutation, named *lde* for lethal dwarfism with epilepsy, was identified as a 13-bp deletion in Exon 9 of the *Wwox* gene resulting in a frame shift mutation causing aberrant amino acid sequences (371–424aa) at the C-terminal of the Wwox protein. Remarkably, *lde/lde* rats displayed phenotypic characteristics similar to *Wwox* KO mice including severe dwarfism and early lethality. The cause of dwarfism is not clear, however, *lde/lde* rats had slightly lower bone density and no osteosarcomas developed during their short life span [27]. Interestingly, *lde/lde* rats displayed significantly higher levels of BUN compared to normal rats [27] consistent with the blood chemistry of our *Wwox* KO mice.

In conclusion, we have generated a novel mouse model with a conditional allele at the *Wwox* locus. We demonstrated that homozygous *Cre*-recombinase mediated deletion of *Wwox* Exon 1 lead to complete ablation of *Wwox* expression. Loss of *Wwox* expression by this approach resulted in a phenotype similar to the previously reported *Wwox* KO generated by conventional techniques making this *Wwox^flox^* strain an important reagent for studying *Wwox* function in normal mouse physiology and tissue specific tumorigenesis.

## Materials and Methods

### Animal Husbandry

Mice used in this were kept in a clean, modified-barrier animal facility, fed regular commercial mouse diet (Harlan Lab., Indianapolis, IN) under controlled light (12L∶12D) and temperature (68–74°F). All animal research was conducted in facilities accredited by the Association for Assessment and Accreditation of Laboratory Animal Care International (AAALAC) at the University of Texas, M.D. Anderson Cancer Center, Science Park Research Division, following international guidelines and all research was approved by the corresponding Institutional bioethics committee (IACUC).

### Gene Targeting and Generation of a *Wwox*-Floxed Allele

The *Wwox* targeting construct was generated using ∼6 Kb of *Wwox* gene obtained from BAC clone 9N99 containing a 5′ portion of the *Wwox* gene from 129/svJ mouse genomic DNA. Primers used for generating the targeting construct were: Right arm forward, 5′-CAAAACCCGGAAACTGGATTACAGC-3′;

Right arm reverse, 5′-CCTAGACTGTGCTGCTCCAA-3′; Left arm forward, 5′-GAGGAGGAGGAGGAGGGAAGTAGAGG-3′; Left arm reverse, 5′-CAGCTACTGGCGGGAAAGGG-3′. First, a ∼4 Kb genomic sequence containing *Wwox* Exon1 and a portion of Intron 1 was PCR amplified and cloned into plasmid pK-11 [28]. pK-11 contains the *neomycin transferase* gene under control of the *phosphoglycerol kinase* promoter (*pgk-neo*) used as the positive-selection element and a single LoxP *Cre*-recombinase recognition site. Another LoxP *Cre*-recombinase recognition site was inserted into *Wwox* Intron 1 in the same orientation as the pK-11 LoxP site in order to “flox” *Wwox* Exon 1 (*Wwox^flox^*) resulting in the plasmid pK-11-*Wwox*-flox. The left arm of the targeting construct was generated by cloning ∼2 Kb of the *Wwox* 5′ upstream sequence into the plasmid pBJ101-DT [22]. Plasmid pBJ101-DT contains the *diphteria toxin A-chain* gene (*DT-A*) used as the negative-selection element. The final targeting construct was generated by transferring the *DT-A* gene and *Wwox* left arm sequences to pK-11-*Wwox*-flox plasmid (Figure 1A).

The *Wwox* target construct was then used by the UT MD Anderson Genetically Engineered Mouse Facility (GEMF) for generation of targeted ES cells and mouse chimeras. ES cell clones were electroporated with the *Wwox* targeting vector that had been linearized by digestion with Not I restriction endonuclease. Transfected ES cells were selected for G418-resistance and individual clones screened by Southern hybridization. Genomic DNA was digested with Bgl II and hybridized to a probe corresponding to a region of *Wwox* Intron 1 outside of the targeting construct sequences (Figure 1A). Correctly targeted ES cells were identified by hybridization to two Bgl II restriction fragments of ∼9 Kb and ∼4 Kb representing the *Wwox^+^* and *Wwox^flox^* alleles, respectively (Figure 1A,B). The hybridization probe was generated by PCR amplification with the following primers: HYB probe reverse, 5′-TCCTTCTGCCAAATCCCGTTATGC-3′, HYB probe forward, 5′-CAAAACTCAAAGTAGAAAGAACAAGG-3′ using 129/svJ mouse genomic DNA as a template. Two correctly targeted ES cell lines were identified from 240 ES cell clones screened.

Injections of targeted ES cells into C57BL/6 blastocysts were performed to generate male chimeras that were mated to C57BL/6 females to test for germline transmission of the *Wwox^flox^* allele. Tail DNA samples from agouti offspring were genotyped by Southern hybridization. *Wwox^+/flox^* offspring were then used as the F_1_ generation for subsequent use. After establishing Mendelian transmission of the *Wwox^flox^* allele genotyping was performed using a multiplex PCR method (Figure 1C,D). The following primers were used for PCR genotyping: Primer A: 5′-ATGGGACGAAACTGGAGCTCAGAA-3′; Primer B: 5′-TCAGCAACTCACTCTGGCTTCAAC-3′; Primer C: 5′-GCATACATTATACGAAGTTATTCGAG-3′.

### Generation of *Wwox* Knockout Mice Using *EIIA-Cre* Mediated Deletion

Female *Wwox^flox/flox^* mice were bred with male mice homozygous for the *Cre*-recombinase gene under the control of the adenovirus *EIIA*-promoter to generate the *Wwox^+/ΔCre^* F_1_ generation. *EIIA-Cre* transgenic mice [23] were obtained from The Jackson Laboratory (Bar Harbor, ME; stock number: 003724). Genotypes were determined by PCR using the oligonucleotide primers: Primer X: 5′-GCCCCCTTTCCCGCCAGTAGCTG-3′; Primer Y: 5′-GACCCAGATCCCCTAATAACG-3′; Primer Z: 5′-GCCCTCAAGAAGAGGCTGCAATTT-3′.

### Western Blot Analysis

Mouse tissues were dissected and flash frozen in liquid nitrogen then crushed into a fine powder with a mortar and pestle. Total protein lysates were made by suspension of crushed tissue with lysis buffer (1% Igepal CA-630, 50 mM Tris-Cl, pH 7.5, 150 mM NaCl, 5 mM EDTA, 10% glycerol) containing protease inhibitors and incubation at 4°C for 30 min. with constant agitation. Insoluble material was then removed by centrifugation at 10,000 x g for 10 min. at 4°C. Protein concentration was determined using the BCA method (Pierce, Rockford, IL). Western blot analysis was performed as previously described [21] using 30 µg of total protein. WWOX protein was detected using a rabbit polyclonal antibody raised against the WWOX WW-domains [24]. The amount of β-actin in each sample was used as an internal control. β-actin was detected using a mouse monoclonal antibody (Sigma Aldrich, St. Louis, MO).

### Mouse Necropsy and Histological Analyses

Mice were euthanized by CO_2_ asphyxiation, organs harvested and immediately weighed using a Mettler AE50 analytical balance.Organs were then fixed in formalin for 24 hours, then 70% ethanol and embedded in paraffin. Tissue sections were stained in H&E or processed for immunostaining. Blood was collected immediately following euthanasia by intracardiac puncture on the left ventricule and allowed to clot at room temperature for 30 minutes. Serum was collected following centrifugation for 10 minutes at 14,000 rpm at 4°C. Blood chemistries were measured using serum from each individual mouse using the Olympus AU400 automated chemistry analyzer. Manual differential and WBC were obtained from feathered blood smears using fresh whole blood from each individual animal.

### Bone Analyses

Limbs were dissected and soft tissue was carefully removed then fixed in formalin for 48 hours followed by 70% ethanol. Microcomputed tomography (micro-cT) was performed at the Baylor College of Medicine MicroCT Core facility. Bone histomorphometric analyses were performed at the UT MD Anderson Bone Histomorphometry Core Laboratory (Director, Dr Nora Navone) following the guidelines established by the American Society for Bone and Mineral Research (ASMBR) histomophometry nomenclature committee.
